# Predicting student academic performance using TabPFN and SHAP: An interpretable machine learning approach

**DOI:** 10.1371/journal.pone.0328634

**Published:** 2026-09-24

**Authors:** Qiang Yin

**Affiliations:** School of Information Science and Engineering, Shandong Normal University, Jinan, Shandong, China; Shanghai Jiaotong University: Shanghai Jiao Tong University, CHINA

## Abstract

Predicting student academic performance is of significant importance for enabling timely educational interventions and improving educational outcomes in secondary education. However, existing predictive models in educational data mining often face the dual challenges of limited interpretability and suboptimal performance on small to medium-sized datasets. This study introduces the Prior-Data Fitted Network (TabPFN) combined with SHapley Additive exPlanations (SHAP) to construct an interpretable machine learning framework for predicting student achievement. Utilizing a publicly available dataset of 4,000 Spanish high school students (SAP-4000), we employed 10-fold cross-validation to train and compare eight machine learning models. The results demonstrate that TabPFN achieves superior predictive performance, with an RMSE of 4.8454, an R^2^ of 0.9216, and an MAE of 3.7950, outperforming traditional models. Comparative analyses across varying data scales indicate that TabPFN maintains high accuracy even when sample sizes are reduced to 500 samples, achieving performance comparable to or better than what traditional models attained at 3,500 samples, exhibiting stronger robustness than commonly used ensemble learning methods such as XGBoost. SHAP analysis reveals that learning behavior and study habit features—specifically weekly study hours, attendance rate, and tutoring status—play a decisive role in predicting academic performance. The proposed framework combines strong predictive performance with interpretability, overcoming the “black-box” limitation prevalent in existing algorithms. It is well-suited for small to medium-scale data modeling tasks in the educational domain, providing technical support for personalized instruction and intervention, while also offering methodological references for future research. An additional case study on a distinct student performance dataset further confirms the model’s robustness and generalizability across different educational prediction tasks.

## Introduction

The digital transformation of education has made the prediction of student academic performance a critical means of enhancing educational quality and enabling personalized interventions. At the secondary education level, students face high-stakes examinations such as university entrance tests, and their academic achievement is influenced by a multitude of interrelated factors. Accurately predicting student performance enables educators to identify at-risk students early and provide targeted support [1]. This study focuses on the traditional in-person secondary education context in Spain, rather than online learning environments.

However, existing research still confronts two major challenges. First, traditional machine learning methods exhibit limited generalization capability in small-sample scenarios. The field of educational data mining continues to rely heavily on traditional methods such as Random Forests and XGBoost. While the performance of these methods typically improves with increasing training data, their predictive accuracy and stability may be constrained in the small-sample scenarios commonly encountered in educational research [2]. Second, the “black-box” problem of high-performance models. Deep learning models and certain ensemble algorithms face a crisis of trust due to their opaque decision-making processes, which is particularly problematic in educational contexts. Studies have shown that when making critical decisions such as academic warnings or personalized recommendations, teachers and students remain cautious about non-transparent model decisions [3,4]. This opacity directly conflicts with the stringent explainability requirements of the education sector, hindering the widespread adoption of machine learning techniques in educational practice.

Notably, TabPFN (Prior-Data Fitted Network), a novel pre-trained model based on the Transformer architecture, offers a new approach to addressing small-sample prediction problems. Pre-trained on millions of synthetic datasets, TabPFN enables fast and accurate predictions in small-sample scenarios and has been validated in domains including medical diagnosis, industrial inspection, and agricultural prediction [5,6]. However, its application in educational research remains unexplored. Given that educational datasets are often limited in size due to practical constraints in data collection, TabPFN’s demonstrated effectiveness in small-sample scenarios makes it a promising candidate for student performance prediction tasks. This study therefore introduces TabPFN to the educational data mining domain and systematically evaluates its performance against established machine learning baselines.

Previous work on academic performance prediction has followed a clear methodological progression. Early studies predominantly relied on statistical methods such as analysis of variance (ANOVA) and regression analysis, establishing foundational associations between student demographic characteristics, study habits, family background, and academic performance [7–9]. Research has consistently demonstrated that factors such as study time, attendance rate, and parental education level are significantly associated with academic outcomes [10]. ANOVA, as a classical statistical method, is frequently employed in educational research to examine the effects of teaching methods, course design, or student characteristics on academic performance [11]. Regression analysis, by modeling the relationship between student behaviors and outcomes, helps identify factors that significantly influence academic performance [12]. Furthermore, cross-lagged models have been used to explore the causal relationship between engagement and performance [13].

With the growth of data scale and computational capacity, supervised learning methods have been widely applied to academic performance prediction, yielding significant improvements in predictive accuracy [14,15]. Supervised learning models are trained using labeled data, adjusting model parameters and structures until they can optimally predict student academic performance [16,17]. Common supervised learning models include Support Vector Machines, Decision Trees, Random Forests, and XGBoost. Support Vector Machines are particularly effective for handling classification problems in high-dimensional feature spaces [18]. For example, Mohd Zaki, Abdul Aziz [19] applied SVM to data from a university learning management system to group students based on academic performance, finding that SVM could effectively capture non-linear patterns in student behavioral features. To overcome the limitations of SVM and other linear models in handling complex non-linear relationships, decision tree methods were proposed, and their performance was further optimized through ensemble methods such as Random Forest [20–22]. Studies have shown that machine learning algorithms can effectively predict student academic performance [23]. Chong, Ibrahim [24] explored the application of machine learning techniques in predicting student engagement and academic performance in online higher education, emphasizing the importance of machine learning in educational prediction research. XGBoost, a gradient-boosting ensemble learning method, has also demonstrated excellent performance in predicting student academic performance [25]. Studies have shown that XGBoost outperforms traditional machine learning models when handling large-scale non-linear data and high-dimensional features [14]. Alruwais and Zakariah [26] compared multiple classifiers including SVM, random forest, and gradient boosting machines, demonstrating that the gradient boosting machine achieved the highest prediction accuracy.

Unlike supervised learning methods, unsupervised learning methods do not rely on labeled data and instead analyze the underlying structure of the data, making them valuable for understanding the complex characteristics of academic performance [27]. Common unsupervised learning techniques include clustering analysis and dimensionality reduction methods. Clustering analysis groups students with similar learning behaviors together, revealing differences in behavioral patterns across different student groups. Studies have shown that clustering analysis can categorize students into high, medium, and low performance groups, with significant differences in behavior and outcomes across groups, providing a basis for personalized teaching interventions [28]. A study using clustering methods on data from an online learning platform found that high-performing student groups consistently showed higher study time, assignment submission frequency, and class participation compared to low-performing groups [29]. Dimensionality reduction methods have significant advantages in handling high-dimensional data and have been widely applied in large-scale data processing tasks [30].

Semi-supervised learning methods combine the advantages of supervised and unsupervised learning, using both labeled and unlabeled data for model training to improve prediction accuracy [31]. In online learning environments, acquiring labeled data typically involves high costs or significant time investment, so semi-supervised learning has unique advantages in these scenarios [32]. For example, Graph Convolutional Networks (GCN) effectively utilize relationships between nodes in graph-structured data to learn more comprehensive student behavioral features. Mubarak, Cao [33] applied a GCN model to analyze student behavioral data from an online learning platform and found that by introducing graph-structured data, GCN could capture latent patterns from a large amount of unlabeled data, significantly improving the accuracy of academic performance prediction.

As data resources continue to accumulate, the machine learning methods discussed above show limitations when faced with massive, high-dimensional, and dynamically changing complex structured data (such as time-series data) [34]. Meanwhile, the development of deep learning offers promise for addressing challenges such as feature extraction difficulty and insufficient pattern recognition accuracy, leading researchers to turn their attention to deep learning technologies [35]. For example, Lumacad and Namoco [36] used student registration and survey data from a private high school in the Philippines, applying a multilayer perceptron (MLP) neural network model for learning style classification, achieving an accuracy of 89.9% on the test set. In the domain of academic performance prediction, Convolutional Neural Networks (CNNs) process student behavioral sequence data through convolution operations, automatically extracting temporal dependence features, thereby effectively predicting student performance. Compared to traditional methods, CNN-Transformer can effectively identify key behavioral patterns in the learning process and handle high-dimensional, complex behavioral data, providing more accurate predictions [37]. Long Short-Term Memory (LSTM) networks, which capture long-term dependencies in data, have been effectively applied to process time-series features in student behavioral data [23].

In summary, machine learning methods have made significant progress in predicting student academic performance, providing new perspectives for personalized teaching and intervention strategies [38,39]. However, despite these achievements, two critical challenges persist. First, traditional machine learning algorithms, including widely-used ensemble methods such as Random Forest and XGBoost, often exhibit limited generalization capability in small-sample scenarios, which are common in educational research due to data collection constraints. While increasing training data generally improves their performance, stable predictions remain difficult to achieve with limited samples [2]. Second, model interpretability remains a significant barrier to practical adoption. Deep learning models and complex ensemble methods operate as “black boxes,” making their decision-making processes opaque to educators and stakeholders. This lack of transparency conflicts with the educational sector’s stringent requirements for explainability, hindering the deployment of machine learning techniques in real-world educational practice [3,4]. To address these challenges, we propose integrating TabPFN—a pre-trained Transformer-based model designed for small-sample tabular data—with the SHAP interpretability framework to achieve both high predictive accuracy and transparent decision-making.

Based on the above background, the research contributions of this paper are as follows:

(1) Based on a publicly available student academic performance dataset, the TabPFN algorithm is systematically compared with seven traditional machine learning models, verifying its effectiveness and robustness on small-sample educational data through repeated random sampling experiments across varying data scales (500–3,500 samples).
(2) To further verify the accuracy and robustness of the TabPFN model in small-scale data prediction, 30 repeated random samplings are conducted across different sample sizes (500–3,500), systematically comparing the performance differences between TabPFN and traditional models such as Random Forest and XGBoost.
(3) The SHAP interpretability framework is integrated with TabPFN to provide both global and local explanations of model predictions, identifying key factors influencing student academic performance (e.g., weekly study hours, attendance rate, tutoring status) and offering actionable insights for personalized teaching interventions.

## Methodology

**Fig 1:**
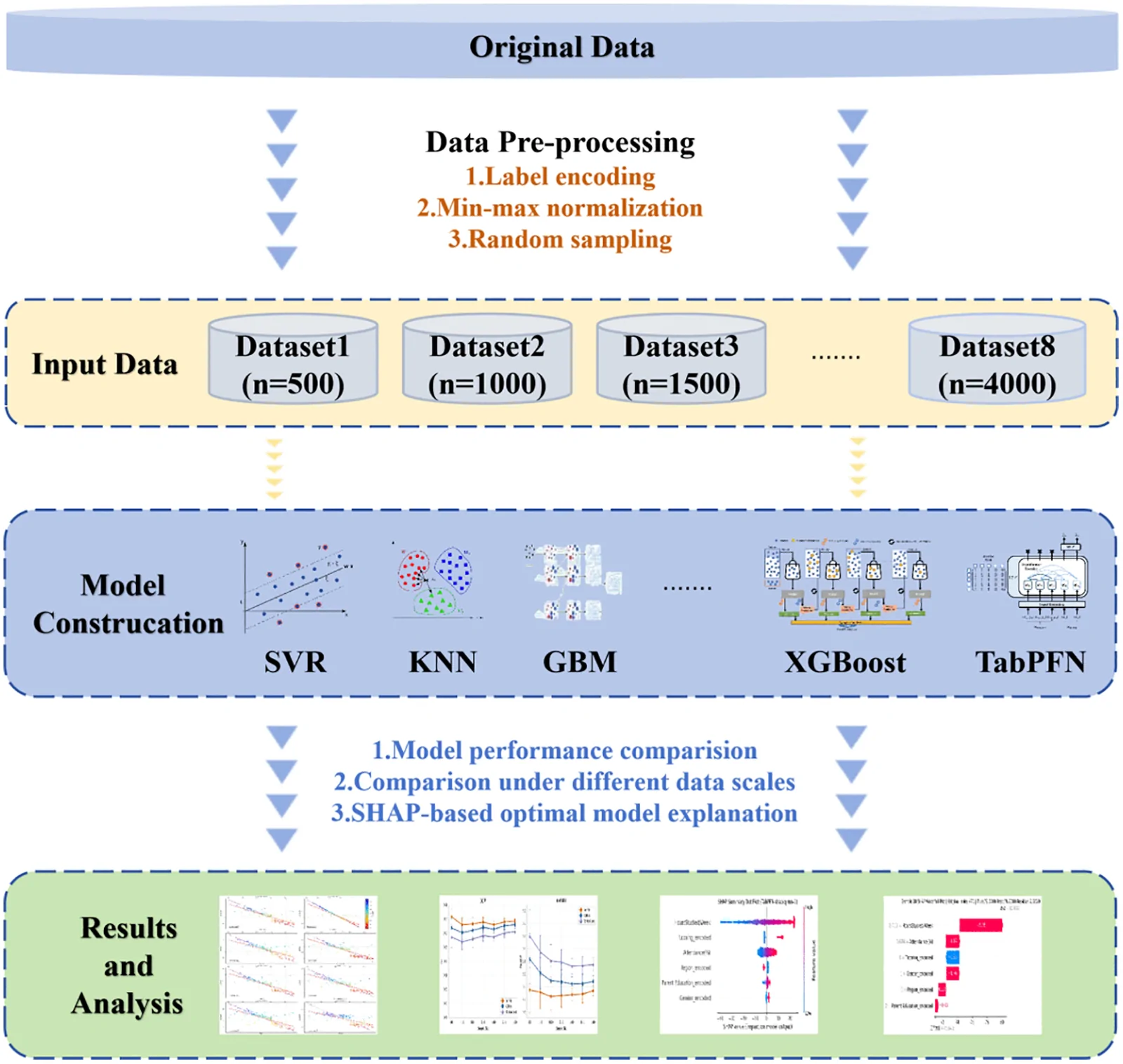
Research Modeling Framework. The diagram illustrates the overall workflow of the study, including data preprocessing, model training with 10-fold cross-validation on eight machine learning models, and model evaluation with SHAP analysis.

Fig 1 illustrates the research framework of this study. The first step involves data preprocessing, which includes label encoding, min-max normalization, and repeated random sampling to prepare the training datasets. Subsequently, eight machine learning models are trained using a 10-fold cross-validation scheme. Finally, the analysis is based on the results of model evaluation, which includes: [1] a comparison of the prediction performance of the eight models on the full dataset; [2] a comparison of model performance across varying data scales (500–3,500 samples) through 30 repeated random samplings, further examining the applicability of TabPFN in small data contexts; and [3] model interpretation and knowledge extraction based on SHAP.

### Data Source and Preprocessing

This study utilizes the “SAP-4000” dataset (Student Academic Performance 4000), sourced from the Kaggle platform. This dataset was originally collected to systematically analyze the multidimensional factors influencing student academic achievement in Spain’s upper secondary education stage (Título de Bachiller), where students are preparing for the university entrance examination (EBAU). The dataset comprises 4,000 anonymized student records collected through structured questionnaires administered prior to final examinations. It is a publicly available, pre-collected dataset that has been anonymized to protect student privacy. Its open accessibility ensures the reproducibility of our results.

The dataset integrates a diverse array of variables, encompassing student demographic information, study habits, educational support mechanisms, and final examination outcomes. Specific features include: student gender (Gender), type of residential area (Region, categorized as Urban/Rural), weekly study hours (HoursStudied/Week, ranging from 0 to 16 hours), attendance rate (Attendance, ranging from 50% to 100%), receipt of additional tutoring support (Tutoring), the highest educational level attained by parents or guardians (Parent Education, categorized as an ordinal variable with four levels: None, Primary, Secondary, Tertiary), and the core outcome variable, final examination score (Exam_Score, ranging from 10 to 100 points). Collectively, these variables construct a multidimensional framework for evaluating academic performance, providing a data foundation for exploring the complex factors that contribute to academic success.

### Descriptive statistics of the data

Table 1 presents the descriptive statistics for all variables in the SAP-4000 dataset. Among the 4,000 students, 51.0% were female, while the remainder were male. In terms of residential area, 60.6% of students resided in urban areas, with rural students constituting the remaining 39.4%. Weekly study hours exhibited considerable variation, with a mean of 9.9 hours (SD = 3.7) and a range spanning from 0 to 16 hours, reflecting diverse study commitments among students. The average attendance rate was 75.2% (SD = 14.5%), with a minimum of 50% and a maximum of 100%. Approximately 30.8% of students reported receiving additional tutoring support.

**Table 1:** Descriptive statistics of numerical data.

Variable	Type	Mean/ Proportion	Std. dev.	Min	Max
Gender (Female)	Categorical	51.0%	—	—	—
Region (Urban)	Categorical	60.6%	—	—	—
HoursStudied/Week	Numerical	9.9	3.7	0	16
Attendance (%)	Numerical	75.2%	14.5	50	100
Tutoring (Yes)	Categorical	30.8%	—	—	—
Parent Education(Secondary)	Categorical	39.7%	—	—	—
Exam_Score	Numerical (Target)	71.1	16.7	17	100

Regarding parental education level, which is commonly used as a proxy for socioeconomic status in educational research, this variable was treated as an ordinal categorical variable with four levels ordered from lowest to highest educational attainment. The distribution was as follows: 8.7% of parents had no formal education, 20.2% had attained primary education, 39.7% had attained secondary education, and 31.4% had attained tertiary education. More than seventy percent of students’ parents had completed at least secondary education, indicating a relatively favorable family educational background in this sample.

The target variable, Exam_Score, ranged from 17 to 100, with a mean of 71.1 (SD = 16.7), indicating substantial variability in student academic outcomes.

**Fig 2:**
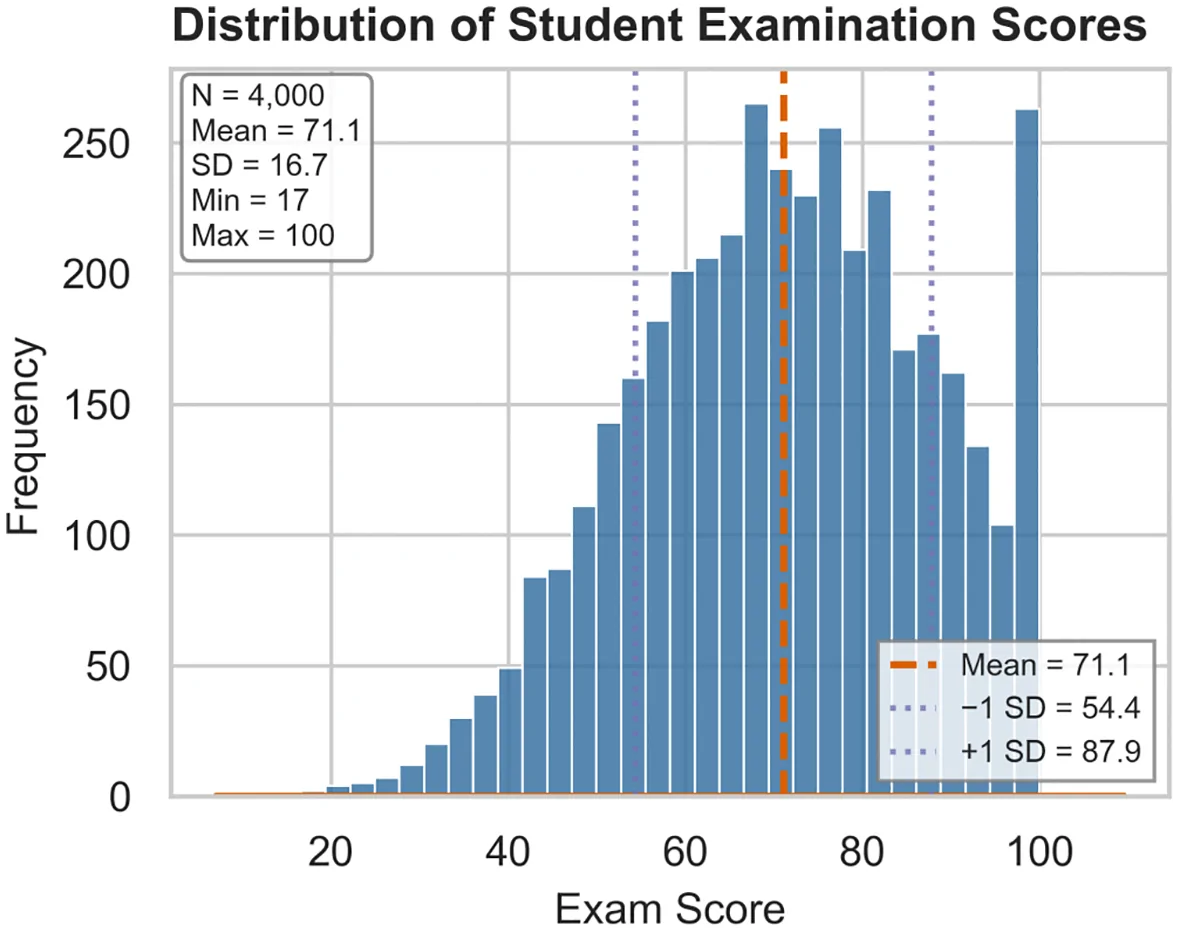
Distribution of Student Examination Scores. Histogram showing the distribution of Exam_Score among 4,000 students, with mean = 71.1 and SD = 16.7.

Fig 2 presents the distribution of the target variable (Exam_Score). The distribution exhibited an approximately normal pattern with a slight left skew, suggesting that a moderate proportion of students performed above the average level, while a smaller number achieved notably lower scores. The mean score was 71.1 (SD = 16.7), with the majority of students scoring between 54.4 and 87.8. This distribution pattern confirms that Exam_Score is suitable for regression-based prediction tasks without requiring substantial transformation.

**Fig 3:**
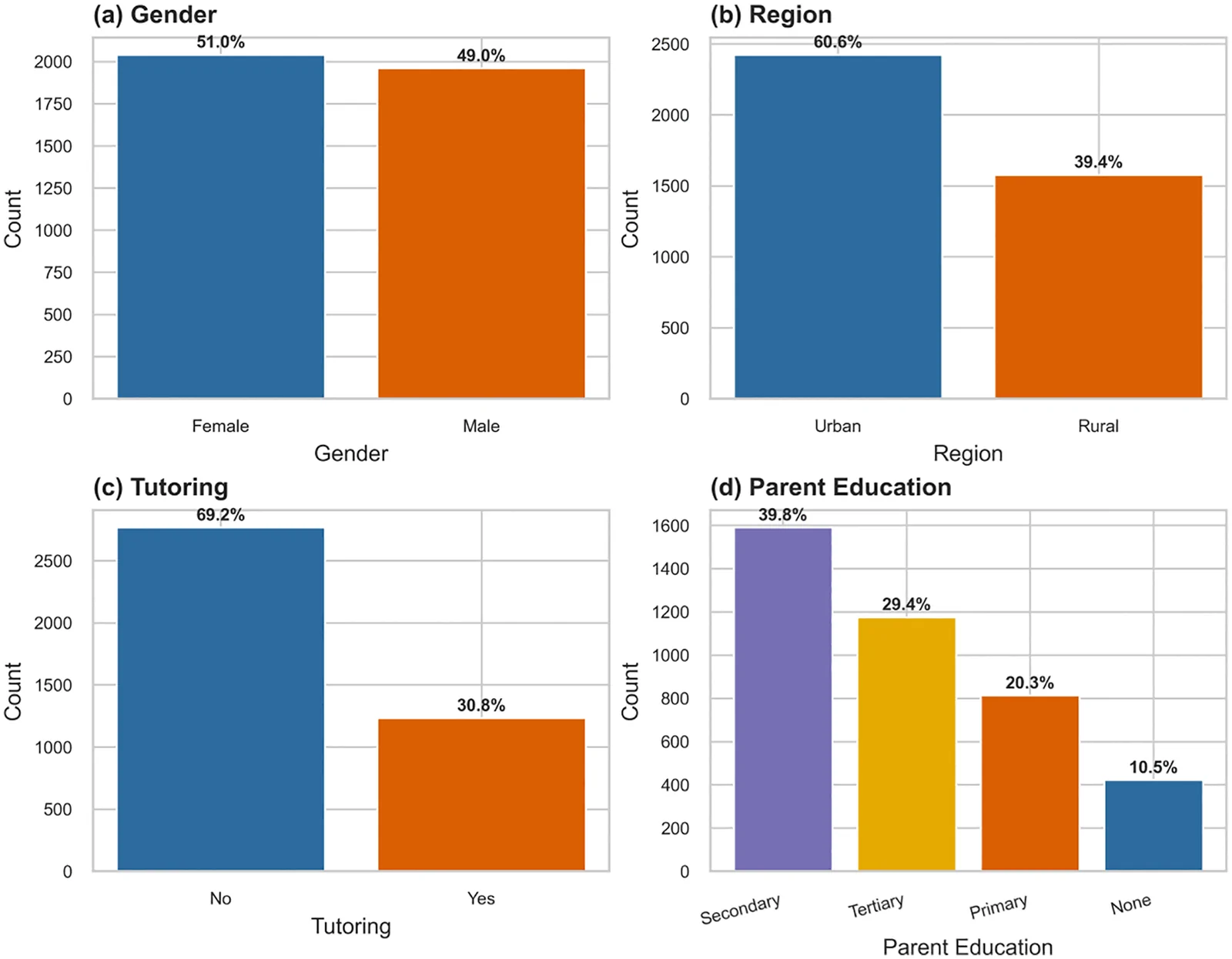
Distribution of Categorical Variables. (a) Gender, (b) Region, (c) Tutoring status, (d) Parental education level.

Fig 3 visualizes the distribution of categorical variables, including gender, region, tutoring status, and parental education level. Gender distribution was relatively balanced (51.0% female vs. 49.0% male). Regarding residential area, urban students (60.6%) outnumbered their rural counterparts (39.4%). The majority of students did not receive tutoring support (69.2%), while 30.8% did. Parental education levels displayed a pyramidal structure, with secondary education being the most common category (39.8%), followed by tertiary (29.4%), primary (20.3%), and no formal education (10.5%). These visualizations provide an intuitive understanding of the sample composition and help contextualize the subsequent predictive modeling results.

### Data preprocessing

All preprocessing procedures described below were applied uniformly to every model in this study to ensure fair comparison. This study employs a systematic data processing pipeline to prepare the raw dataset for model training and evaluation. The SAP-4000 dataset was first examined for data quality issues. No missing values were detected across any features, and no irrelevant identifier variables were present in the dataset. Consequently, the preprocessing procedure focused on feature encoding, normalization, and sampling.

Categorical variables were encoded into numerical representations to enable model processing. The dataset contains four categorical features: Gender (Male/Female), Region (Urban/Rural), Tutoring (Yes/No), and Parent Education (None/Primary/Secondary/Tertiary). For the binary categorical variables (Gender, Region, and Tutoring), label encoding was applied, mapping each category to 0 or 1. For Parent Education, which is an ordinal categorical variable with a natural ordering from lower to higher educational attainment, ordinal encoding was employed with the mapping None = 0, Primary = 1, Secondary = 2, and Tertiary = 3. This approach preserves the inherent order of educational levels while enabling the models to process the feature numerically, avoiding the dimensionality expansion that would result from one-hot encoding.

All continuous variables underwent min-max normalization to eliminate the influence of different scales on the analysis results. The numerical features subjected to normalization included HoursStudied/Week (range 0–16), Attendance(%) (range 50–100). This transformation maps all values to the [0, 1] interval, ensuring that features with larger numerical ranges do not disproportionately influence model training.

We examined the data for outliers using boxplot (IQR method) and z-score (3σ) analyses. No extreme outliers were detected in the numerical features HoursStudied/Week or Attendance(%). For the target variable Exam_Score, a small number of low-scoring students (6 out of 4,000, 0.15%) were identified as statistical outliers. These cases represent genuine instances of low academic performance rather than data errors. Given that identifying such students is central to the purpose of this study—predicting at-risk students for early intervention—these cases were retained in the dataset without removal.

To comprehensively evaluate model performance across varying data scales, we implemented a repeated random sampling strategy. The full dataset of 4,000 samples was retained as the baseline condition representing the maximum available data scale. Seven subsets of varying sizes were then generated from the full dataset: 500, 1,000, 1,500, 2,000, 2,500, 3,000, and 3,500 samples. For each subset size, 30 independent random draws were performed without replacement from the full dataset. Each draw was stratified by the target variable (Exam_Score) to maintain distributional consistency across all subsets. For each repetition, a distinct random seed was used (starting from 42 and incrementing by 1 for each subsequent repetition) to ensure the independence of each sampling iteration.. This repeated sampling design allows systematic evaluation of model performance across different data scales, with particular attention to TabPFN’s performance in small-sample scenarios relative to traditional ensemble methods. It also enables the quantification of performance variability across multiple subsamples, thereby providing more reliable estimates of model robustness and generalizability than single-draw evaluations would permit.

### Multicollinearity analysis

To assess multicollinearity among the predictor variables after preprocessing, we calculated the Variance Inflation Factor (VIF) for each feature. VIF quantifies the extent to which the variance of a regression coefficient is inflated due to correlations with other predictors. A VIF value exceeding 5 or 10 is commonly interpreted as indicating moderate to severe multicollinearity that may affect model interpretability and stability.

As presented in Table 2, all features exhibited VIF values close to 1.00, ranging from 1.0005 to 1.0016, well below the conventional threshold of 5. The highest VIF was observed for Tutoring_encoded (VIF = 1.0016), while the lowest was observed for Parent Education_encoded (VIF = 1.0005). These results confirm that no significant multicollinearity exists among the predictor variables in the SAP-4000 dataset, and thus all features were retained for model training.

**Table 2:** Variance inflation factor (VIF) for predictor variables.

Feature	VIF
Tutoring_encoded	1.0016
Gender_encoded	1.0011
Attendance(%)	1.0010
HoursStudied/Week	1.0007
Region_encoded	1.0007
Parent Education_encoded	1.0005

### Model Training

#### Model Overview.

This study selected eight machine learning models commonly used in educational data prediction for comparison, including Support Vector Regression (SVR) [40], K-Nearest Neighbors Regression (KNN) [41], Gradient Boosting Regression (GBM) [42], Random Forest (RF) [43], Ridge Regression [44], XGBoost (XGB) [45], LightGBM (LGB) [46] and TabPFN [47]. The seven baseline models were selected according to three criteria. First, they represent the most commonly used machine learning methods in educational data mining literature, ensuring that our comparison is grounded in established practices. Second, they cover diverse algorithmic families: a linear model (Ridge Regression), an instance-based learner (K-Nearest Neighbors, KNN), a kernel-based method (Support Vector Regression, SVR), and tree-based ensemble methods including Random Forest (RF), Gradient Boosting Machine (GBM), XGBoost (XGB), and LightGBM (LGB), which represent the current state-of-the-art for tabular data prediction and have been extensively validated in educational prediction tasks. Third, this selection covers both simpler models (Ridge, KNN) and more complex ones (ensemble methods), allowing us to assess TabPFN’s relative performance across different levels of model complexity. TabPFN is a pre-trained model based on the Transformer architecture, specifically designed for small-sample tabular data. It leverages prior knowledge for rapid inference and maintains high predictive performance even with limited data. In recent years, TabPFN has demonstrated outstanding performance in fields such as healthcare and industry, but its application in educational research has not yet been fully explored. The latest release, version 2 [48], further enhances classification performance and introduces new mechanisms for regression tasks.

### Experimental setup

For data partitioning, we employed random stratified sampling to divide the original dataset into a training-validation set (80%) and an independent test set (20%), ensuring a balanced distribution of the target variable. This 80/20 split is consistent with standard practice in machine learning for datasets of this size, providing a sufficiently large training set (approximately 3,200 samples) for stable model training and 10-fold cross-validation, while retaining a test set of 800 samples that is large enough to yield reliable performance estimates. To comprehensively evaluate model performance and mitigate overfitting, we implemented a two-stage validation strategy. First, a 10-fold cross-validation scheme was applied to the training-validation set for model training and hyperparameter tuning. Second, the held-out independent test set (20%) was used for the final model evaluation and statistical comparison. This approach maximizes the utilization of limited data while providing robust estimates of model generalization performance.

To evaluate model performance across varying data scales, we generated seven subsets from the full dataset with sample sizes of 500, 1,000, 1,500, 2,000, 2,500, 3,000, and 3,500. For each subset size, 30 independent random draws were performed without replacement. For each repetition, a distinct random seed was used (starting from 42 and incrementing by 1 for each subsequent repetition) to ensure the independence of each sampling iteration and full reproducibility.

We conducted comparative experiments using multiple machine learning regression models. For all models, hyperparameter optimization was performed using grid search coupled with 10-fold cross-validation on the training-validation set, with negative mean squared error as the scoring metric. The detailed hyperparameter search spaces and the optimal configurations identified for each model are provided in S1 Table. For TabPFN, the search was limited to the n_estimators parameter [8,16,24,32], with the optimal configuration identified as n_estimators = 32. Traditional models were tuned across broader parameter spaces to ensure a fair comparison. The optimized models were then evaluated on the held-out test set.

All experiments were conducted on a cloud-based workstation equipped with an Intel Xeon Platinum 8470Q CPU (17 vCPUs), 60 GB RAM, and an NVIDIA GPU with 32 GB VRAM. The experiments were implemented in Python 3.10 using scikit-learn (version 1.7.2) for traditional models, XGBoost (version 3.2.0), LightGBM (version 4.6.0). Training and inference times for each model are reported in S2 Table.

### Model Evaluation

This study adopts a standard evaluation metric system for regression tasks, including key metrics such as Root Mean Square Error (RMSE), Coefficient of Determination (R^2^), and Mean Absolute Error (MAE).

Root Mean Square Error (RMSE) is the square root of the mean of the squared differences between predicted values and actual values, defined as the following formula:


$$\begin{array}{c} RMSE=\sqrt{\frac{\text{1}}{N}\sum_{i=1}^{N}(y_{i}-{\hat{y}}_{i}{)}^{\text{2}}} \end{array}$$ (1)

Where $y_{i}$ represents the actual values, ${\hat{y}}_{i}$ represents the predicted values, and N represents the sample size. RMSE is more sensitive to larger errors, so in prediction models, a smaller RMSE value indicates better model performance.

The Coefficient of Determination (R-squared, R^2^), also known as the goodness of fit, measures the model’s ability to explain the variance in the observed data. Its formula is:


$$\begin{array}{c} R^{\text{2}}=1-\frac{\sum_{i=1}^{N}(y_{i}-{\hat{y}}_{i}{)}^{\text{2}}}{\sum_{i=1}^{N}(y_{i}-{\overset{―}{y}}_{i}{)}^{\text{2}}} \end{array}$$ (2)

where $\overset{―}{y}$ is the mean of the actual values. The value of R^2^ ranges from [0, 1], with a value closer to 1 indicating a better fit of the model.

Mean Absolute Error (MAE) is the average of the absolute errors between the predicted values and the actual values. Its formula is:


$$\begin{array}{c} MAE=\frac{\text{1}}{N}\sum_{i=1}^{N}|y_{i}-{\hat{y}}_{i}| \end{array}$$ (3)

MAE treats all errors equally, without emphasizing any particular size of errors, and therefore is less sensitive to outliers. A smaller MAE value indicates better model predictive performance.

The three evaluation metrics were selected according to the following rationale. RMSE was chosen for its sensitivity to large errors, which is particularly important for identifying at-risk students who deviate significantly from expected performance—underestimating a struggling student’s risk is more consequential than small prediction errors. R^2^ was selected to measure the proportion of variance explained by the model, providing an intuitive and standardized measure of overall fit that facilitates comparison with prior studies. MAE was included for its interpretability in the original scale of the target variable (exam scores), offering practitioners a straightforward understanding of average prediction error in points. Together, these three metrics provide complementary perspectives: RMSE emphasizes large errors, MAE summarizes typical errors, and R^2^ quantifies explanatory power.

To statistically validate performance differences between models and to assess both the uncertainty and the practical significance of the results, we employed three complementary approaches:

(1) Bootstrap Confidence Intervals: For each model, we calculated 95% confidence intervals for all performance metrics (RMSE, MAE, R^2^) using 1,000 bootstrap iterations, each drawing samples with replacement from the independent test set (20% of the full dataset, approximately 800 samples). This non-parametric approach provides robust interval estimates without distributional assumptions. These intervals reflect the variability of model performance on unseen data and are reported alongside the point estimates.
(2) Paired t-tests: To assess whether TabPFN’s performance superiority was statistically significant, we conducted two-tailed paired t-tests comparing its cross-validated RMSE scores against each baseline model. The t-tests were performed on the 10 cross-validation folds. The null hypothesis was that there was no difference in RMSE between TabPFN and the baseline model. To complement the t-tests and address the small number of folds (k = 10), we also performed Wilcoxon signed-rank tests as a non-parametric alternative. The results of both tests were consistent (Wilcoxon all p < 0.01), and we report the t-test results in the main text for interpretability, with the full Wilcoxon results provided in S3 Table.
(3) Effect Size Calculation: In addition to statistical significance, we computed Cohen’s d effect sizes to quantify the magnitude of performance differences. Cohen’s d was calculated as the mean difference in RMSE divided by the pooled standard deviation, with thresholds of 0.2, 0.5, and 0.8 indicating small, medium, and large effects, respectively.

Additionally, to assess the generalizability of the proposed framework beyond the primary dataset, we conducted an additional case study using a publicly available student performance dataset (https://www.kaggle.com/datasets/rkiattisak/student-performance-in-mathematics/data). This dataset captures demographic and socioeconomic factors (e.g., gender, parental education, lunch type) alongside test preparation behavior and final exam scores in mathematics, reading, and writing. It was selected because it represents a distinct prediction task with a different feature structure from the SAP-4000 dataset, providing a stringent testbed for evaluating whether TabPFN’s advantages generalize across diverse educational contexts and data modalities.

### Model interpretation based on SHAP

To elucidate the decision-making mechanisms of the TabPFN model, we employed the shapiq library, which provides a dedicated TabPFNExplainer. Unlike traditional imputation-based explainers, the TabPFNExplainer adopts a remove-and-recontextualize strategy: for each feature subset, it removes the corresponding feature columns from both the training and test data and re-contextualizes the TabPFN model on the reduced dataset. This approach is inherently faithful to TabPFN’s in-context learning paradigm and eliminates the need for approximating missing features with background samples. The SHAP values were computed for all test set samples (20% of the dataset, n = 800) to ensure that the interpretations reflect the model’s behavior on unseen data.

## Experimental results

### Model performance comparison

To evaluate the predictive performance of all models, we first conducted a comparative experiment using the complete SAP-4000 dataset (4,000 samples). The performance comparison results of each model are shown in Table 3.

**Table 3:** The performance comparison results of each model.

Model	RMSE (95% CI)	MAE (95% CI)	R² (95% CI)
SVR	4.9536 [4.7193, 5.2233]	3.9229 [3.7177, 4.1404]	0.9180 [0.9071, 0.9282]
Ridge	4.9563 [4.7220, 5.2080]	3.9294 [3.7338, 4.1308]	0.9179 [0.9062, 0.9271]
XGBoost	5.0102 [4.7540, 5.2605]	3.9737 [3.7754, 4.1948]	0.9161 [0.9045, 0.9262]
LightGBM	5.0352 [4.7592, 5.2845]	3.9927 [3.7874, 4.1974]	0.9153 [0.9037, 0.9259]
GBM	5.0517 [4.7800, 5.3124]	4.0113 [3.8057, 4.2204]	0.9147 [0.9027, 0.9248]
RandomForest	5.3081 [5.0474, 5.5819]	4.1806 [3.9681, 4.4077]	0.9059 [0.8917, 0.9163]
KNN	5.7851 [5.5033, 6.0993]	4.6231 [4.3770, 4.8656]	0.8882 [0.8756, 0.8994]
**TabPFN**	**4.8454 [4.5940, 5.0934]**	**3.7950 [3.5889, 4.0017]**	**0.9216 [0.9099, 0.9306]**

In terms of RMSE, the TabPFN model achieved the lowest value (4.8454, 95% CI: [4.5940, 5.0934]), indicating the smallest difference between predicted and actual values and the highest predictive accuracy. In contrast, the KNN model exhibited the highest RMSE (5.7851, 95% CI: [5.5033, 6.0993]), suggesting greater prediction errors and a weaker ability to capture underlying data trends. Other models such as SVR (4.9536), Ridge (4.9563), and GBM (5.0517) performed better than KNN but were still outperformed by TabPFN.

The TabPFN model also attained the highest R^2^ value (0.9216, 95% CI: [0.9099, 0.9306]), explaining approximately 92.2% of the variance in the data, which confirms its superior explanatory power. By comparison, the KNN model showed the lowest R^2^ (0.8882, 95% CI: [0.8756, 0.8994]), indicating a relatively poor fit compared to other models. Traditional tree-based models such as XGBoost (0.9161), LightGBM (0.9153), and Random Forest (0.9059) also demonstrated strong performance but remained below TabPFN.

Regarding error metrics, TabPFN achieved the lowest MAE (3.7950, 95% CI: [3.5889, 4.0017]), further underscoring its accuracy in point prediction. The KNN model again showed the highest MAE (4.6231, 95% CI: [4.3770, 4.8656]), reflecting larger absolute errors.

A deeper analysis of performance differences reveals that traditional tree models (XGBoost, LightGBM, GBM, Random Forest), while generally outperforming KNN and SVR, still lag behind TabPFN. This is likely attributable to their dependence on larger datasets to establish stable decision boundaries. In contrast, TabPFN leverages the prior knowledge transfer capabilities of the Transformer architecture, enabling robust feature extraction and pattern recognition even under small-sample conditions. Notably, TabPFN’s MAE (3.7950) was consistently lower than all baseline models, indicating that its predictions exhibit smaller absolute deviations from actual values. This characteristic is particularly valuable for educational applications such as academic early-warning systems and personalized recommendations, where high predictive precision is essential.

**Fig 4:**
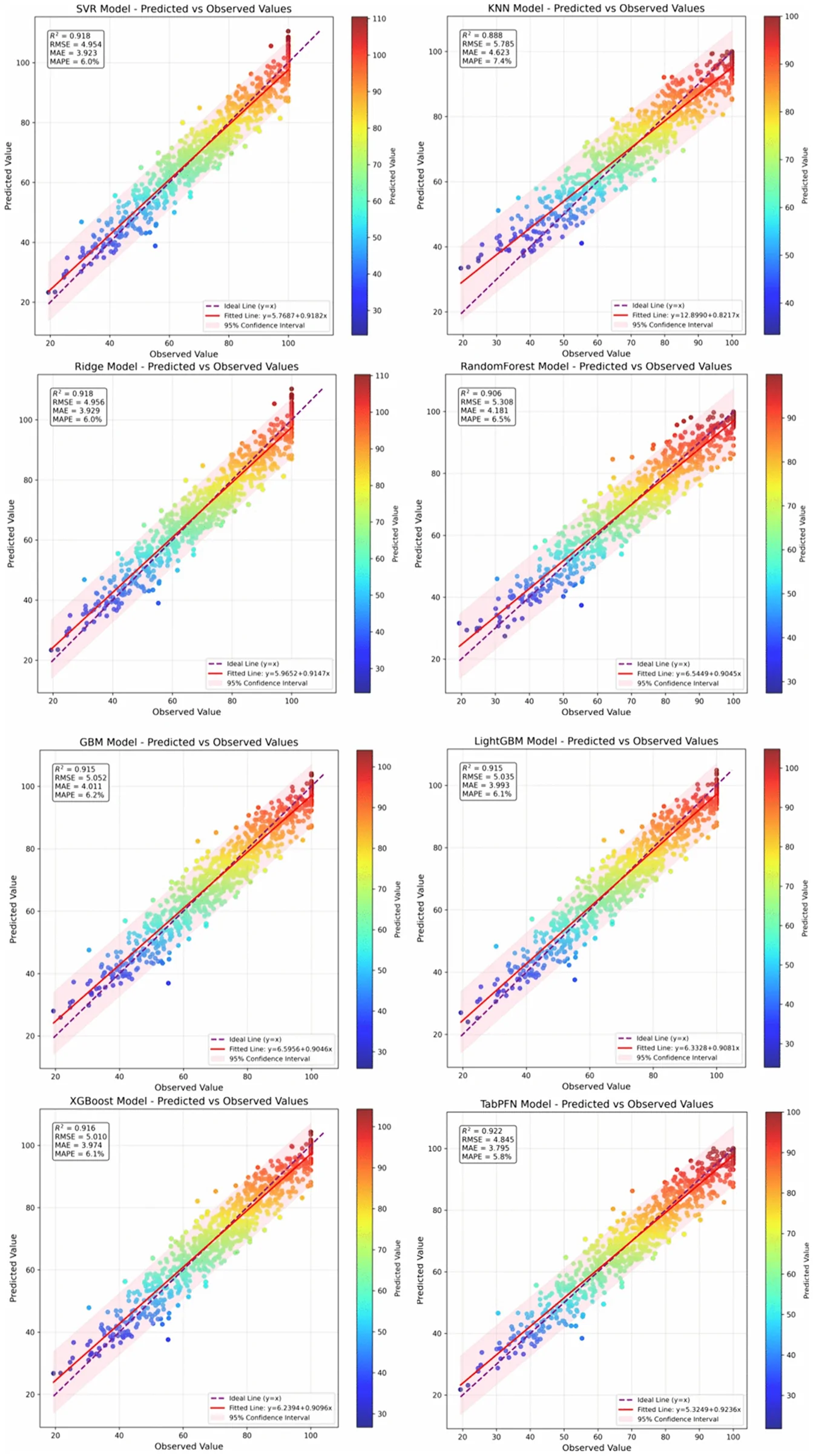
Comparison of Prediction Performance Across 8 Machine Learning Models. Scatter plots of predicted vs. observed values for each model. The red solid line represents the fitting line; the purple dashed line represents the ideal y = x line; the light pink shaded area indicates the 95% confidence interval.

In order to more intuitively demonstrate the predictive performance of the model, we further plotted scatter plots of the predicted values and observed values of eight models based on Table 3. In Fig 4, the horizontal axis represents the observed values and the vertical axis represents the predicted values of the model. The red solid line represents the fitting line, indicating the degree of fit of the model to the data. The purple dashed line represents the ideal situation where the predicted value is equal to the observed value. The light pink shaded area represents the 95% confidence interval, reflecting the uncertainty range of the predicted values.

To determine whether TabPFN’s performance advantage was statistically significant, we conducted two-tailed paired t-tests comparing its cross-validated RMSE scores with those of each baseline model. As presented in Table 4, TabPFN significantly outperformed all seven comparison models (all p < 0.001). The RMSE differences ranged from 0.1038 (vs Ridge) to 0.9994 (vs KNN), all in favor of TabPFN. Corresponding effect sizes, quantified by Cohen’s d, were consistently large (d = −1.617 to −7.068), exceeding the conventional threshold for large effects (d = 0.8). These results provide strong statistical evidence supporting TabPFN’s superiority in predicting academic performance.

**Table 4:** Results of paired t-tests comparing TabPFN with baseline models (two-tailed tests based on cross-validated RMSE scores).

Comparison	RMSE Diff	t-statistic	p-value (two-tailed)	Cohen’s d	Effect Size	Sig
TabPFN vs SVR	0.1039	−5.049	<0.001	−1.683	Large	***
TabPFN vs KNN	0.9994	−21.205	<0.001	−7.068	Large	***
TabPFN vs Ridge	0.1038	−4.850	<0.001	−1.617	Large	***
TabPFN vs GBM	0.1414	−8.871	<0.001	−2.957	Large	***
TabPFN vs RF	0.3738	−8.782	<0.001	−2.927	Large	***
TabPFN vs XGB	0.1292	−6.934	<0.001	−2.311	Large	***
TabPFN vs LGB	0.1310	−9.136	<0.001	−3.045	Large	***

### Impact of data size

**Fig 5:**
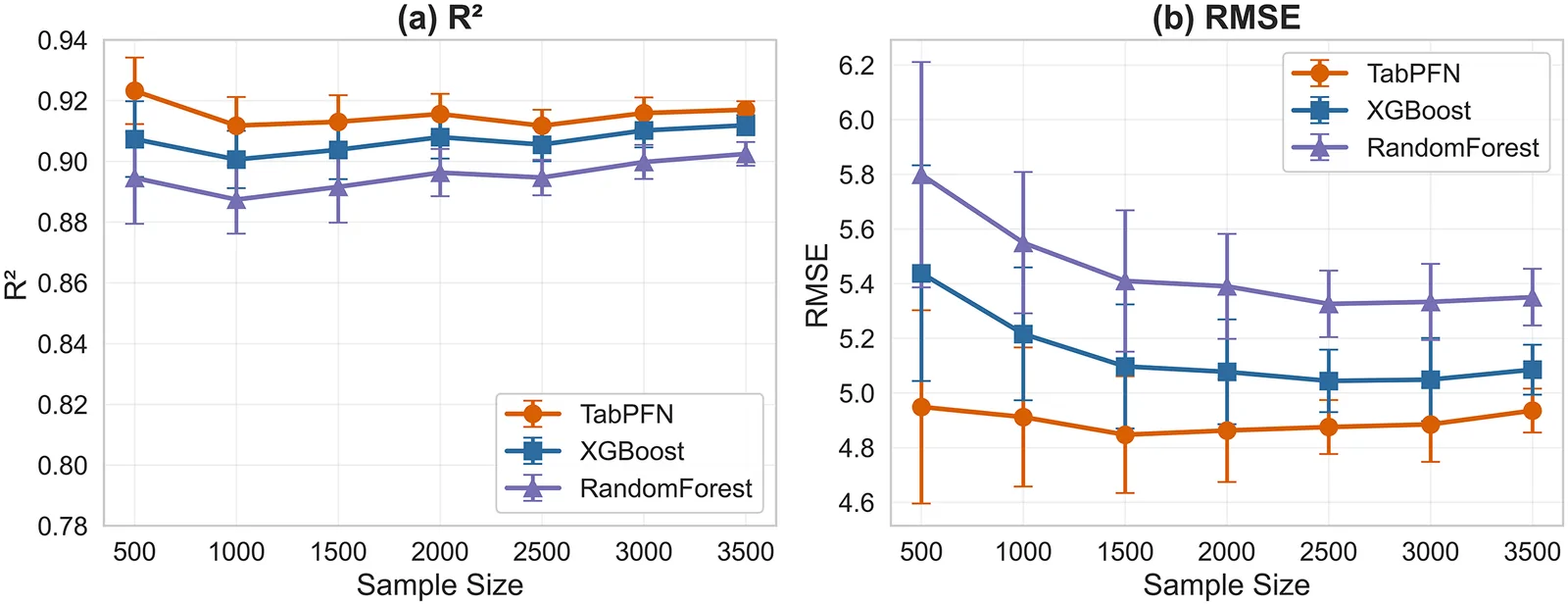
Impact of Sample Size on Model Performance. (a) R^2^ values and (b) RMSE values across different sample sizes (500–3,500). Results are averaged over 30 independent random samplings; error bars represent ±1 standard deviation.

To evaluate the impact of sample size on model performance and further examine the applicability of the TabPFN algorithm for small-data scenarios, we conducted a systematic comparative experiment across varying data scales. Based on the previous analysis, tree-based models (including Random Forest and XGBoost), which are known for their good performance and are commonly used in educational data mining, were selected as the baseline models for comparison. For each subset size (500, 1,000, 1,500, 2,000, 2,500, 3,000, and 3,500 samples), 30 independent random draws were performed, with the random seed fixed at 42 to ensure reproducibility. Fig 5 presents the mean RMSE and R^2^ values across the 30 repetitions for each model and subset size, with error bars indicating ±1 standard deviation to illustrate the variability of model performance across different data splits.

As shown in Fig 5(a), TabPFN achieves relatively stable R^2^ values across all sample sizes, ranging from approximately 0.912 to 0.923. At 500 samples, TabPFN reaches an R^2^ of approximately 0.923, compared to approximately 0.907 for XGBoost and 0.895 for Random Forest. As sample size increases, XGBoost and Random Forest show gradual improvement, reaching approximately 0.912 and 0.902 respectively at 3,500 samples, while TabPFN maintains consistent performance with R^2^ values above 0.91 across all scales.

The performance trends reveal different patterns between TabPFN and the traditional ensemble methods. XGBoost and Random Forest exhibit steady improvement with increasing data, with R^2^ gains of approximately 0.005 and 0.008 respectively from 500 to 3,500 samples. In contrast, TabPFN shows rapid performance saturation, achieving near-optimal results with as few as 500 samples and exhibiting limited additional gains at larger sample sizes.

The RMSE results in Fig 5(b) show similar patterns. TabPFN maintains relatively stable RMSE values across all sample sizes, ranging from approximately 4.86 to 4.94. XGBoost and Random Forest show gradual decreases in RMSE as sample size increases, from approximately 5.44 to 5.08 and 5.80 to 5.35 respectively. At 500 samples, TabPFN achieves an RMSE of approximately 4.95, which is comparable to or slightly better than XGBoost’s performance at larger sample sizes.

Regarding performance stability, the error bars in Fig 5 indicate that TabPFN exhibits relatively small variability across the 30 repetitions compared to the baseline models, particularly at smaller sample sizes. The standard deviations for TabPFN’s R^2^ across all sample sizes range from approximately 0.003 to 0.011, which are generally smaller than those observed for Random Forest (0.004 to 0.022) and XGBoost (0.003 to 0.012). This suggests that TabPFN’s performance may be less sensitive to variations in training data composition, though further investigation would be needed to confirm this observation.

It is worth noting that while TabPFN demonstrates competitive performance at small sample sizes, the differences between models become less pronounced as sample size increases. At 3,500 samples, the R^2^ values for TabPFN, XGBoost, and Random Forest converge to approximately 0.917, 0.912, and 0.902 respectively. This convergence suggests that the advantage of TabPFN is most relevant in data-scarce scenarios, while traditional ensemble methods can achieve comparable performance given sufficient data.

In summary, the multi-scale analysis with repeated random sampling suggests that TabPFN offers competitive performance in small-sample educational scenarios, achieving relatively high accuracy with as few as 500 samples. However, the performance gap between TabPFN and traditional ensemble methods narrows as sample size increases, indicating that the choice of model may depend on the specific data availability constraints of each application. These findings highlight the potential utility of TabPFN for educational research contexts where data collection is limited, while also acknowledging that traditional methods remain viable alternatives when sufficient data are available.

### Additional case study on a different student performance dataset

Additionally, to assess the generalizability of the proposed framework beyond the primary dataset, we conducted an additional case study using a publicly available student performance dataset sourced from Kaggle (https://www.kaggle.com/datasets/rkiattisak/student-performance-in-mathematics/data). This dataset contains information on the performance of high school students in mathematics, including their grades and demographic information, collected from three high schools in the United States. It comprises the following variables: gender, race/ethnicity, parental level of education, lunch type (indicating whether the student receives free or reduced-price lunch), test preparation course completion, and scores on standardized mathematics, reading, and writing tests. This dataset was selected because it represents a distinct prediction task with a different feature structure from the SAP-4000 dataset—focusing on demographic and socioeconomic factors rather than study habits and learning behaviors—providing a stringent testbed for evaluating whether TabPFN’s advantages generalize across diverse educational contexts and data modalities.

Consistent with the main experiment, TabPFN achieved the lowest RMSE (0.0591), the lowest MAE (0.0483), and the highest R^2^ (0.8858). Paired t-tests comparing TabPFN with the best-performing baselines (Ridge and SVR) on this dataset showed that the differences were not statistically significant (p = 0.1062 and p = 0.0689, respectively), indicating that TabPFN performs comparably to these two models on this dataset. However, TabPFN significantly outperformed the other five baseline models (GBM, XGBoost, LightGBM, RandomForest, and KNN), with paired t-test p-values all below 0.005. These results suggest that TabPFN’s predictive advantages generalize to a distinct educational prediction task with a different feature structure, performing at least as well as the strongest baselines and significantly better than most ensemble methods (Table 5).

**Table 5:** Model Performance on the Additional Student Exams Dataset.

Model	RMSE (95% CI)	MAE (95% CI)	R² (95% CI)
SVR	0.0601 [0.0547, 0.0647]	0.0495 [0.0447, 0.0540]	0.8820 [0.8513, 0.9066]
KNN	0.0781 [0.0702, 0.0858]	0.0601 [0.0535, 0.0667]	0.8005 [0.7595, 0.8366]
Ridge	0.0600 [0.0548, 0.0651]	0.0496 [0.0448, 0.0546]	0.8822 [0.8507, 0.9048]
RandomForest	0.0650 [0.0585, 0.0707]	0.0527 [0.0470, 0.0580]	0.8619 [0.8214, 0.8894]
GBM	0.0661 [0.0603, 0.0722]	0.0528 [0.0476, 0.0584]	0.8570 [0.8172, 0.8850]
LightGBM	0.0629 [0.0570, 0.0690]	0.0497 [0.0446, 0.0551]	0.8707 [0.8349, 0.8981]
XGBoost	0.0628 [0.0572, 0.0682]	0.0502 [0.0454, 0.0554]	0.8711 [0.8365, 0.8962]
**TabPFN**	**0.0591 [0.0539, 0.0638]**	**0.0483 [0.0435, 0.0530]**	**0.8858 [0.8563, 0.9084]**

The successful replication of TabPFN’s leading performance on this independent dataset, despite its different feature structure, provides further evidence of the algorithm’s robustness. While the absolute performance metrics are high for all models (R^2^ > 0.85 for top models), TabPFN consistently achieves the highest R^2^ and lowest error rates. This suggests that the benefits of TabPFN’s meta-learning and Transformer architecture may extend beyond the specific feature set of our main study. However, we acknowledge that these results should be interpreted with caution, as this additional case study evaluates a different prediction task with a different set of features. Future work should systematically examine TabPFN’s generalizability across a wider range of educational datasets and task formulations.

### Interpretability analysis for actionable educational insights

To elucidate the decision-making mechanisms of the TabPFN model and extract actionable educational insights, we employed SHAP (SHapley Additive exPlanations) for interpretability analysis [49]. SHAP, grounded in cooperative game theory, quantifies the contribution of each feature to individual predictions by calculating Shapley values. This approach provides both global interpretability (overall feature importance) and local interpretability (explanations for specific predictions).

In our implementation, we utilized the TabPFNExplainer from the shapiq library, which provides a dedicated explainer for TabPFN models. Unlike traditional imputation-based explainers such as KernelExplainer, the TabPFNExplainer adopts a “remove-and-recontextualize” strategy: for each feature subset, it removes the corresponding feature columns from both the training and test data and re-contextualizes the TabPFN model on the reduced dataset [50]. This approach is inherently faithful to TabPFN’s in-context learning paradigm and eliminates the need for approximating missing features with background samples. The SHAP values were computed for all test set samples (20% of the dataset, n = 800), ensuring that the interpretations reflect the model’s behavior on unseen data.

The analysis proceeded through multiple complementary visualizations

Global Feature Importance: We calculated the mean absolute SHAP values across all test samples to identify which features most consistently influence exam score predictions.

Summary Plots: We generated dot summary plots to visualize the distribution of SHAP values for each feature, revealing not only their importance but also the direction (positive/negative) and magnitude of their effects on the predicted outcomes.

Individual Explanation Waterfall Plots: For each test sample, we created detailed waterfall plots that decompose the model’s prediction into contributions from individual features. These visualizations make transparent how specific feature values (e.g., HoursStudied/Week, Attendance, Tutoring status) combine to produce the final exam score prediction.

The entire SHAP analysis was conducted exclusively on the held-out test set (20% of data, n = 800), ensuring that interpretations reflect the model’s generalization behavior rather than potential overfitting to training patterns. This approach maintains mathematical rigor while providing educators and researchers with both macroscopic insights (which student behaviors and study habits matter most) and microscopic transparency (how specific student profiles lead to particular performance predictions).

It is important to acknowledge that SHAP analysis, like other post-hoc interpretability methods, provides approximate explanations that may be sensitive to the choice of explainer and the underlying model. In this study, we opted for the TabPFNExplainer specifically designed for TabPFN models to ensure methodological consistency. However, the stability of SHAP values across different explainer configurations and the potential influence of feature correlations on importance rankings warrant consideration when interpreting the results. Despite these limitations, SHAP remains one of the most widely adopted tools for interpreting machine learning models, and our use of a test-set-only analysis strategy enhances the reliability of the explanations for practical deployment scenarios.

### Global explanation

**Fig 6:**
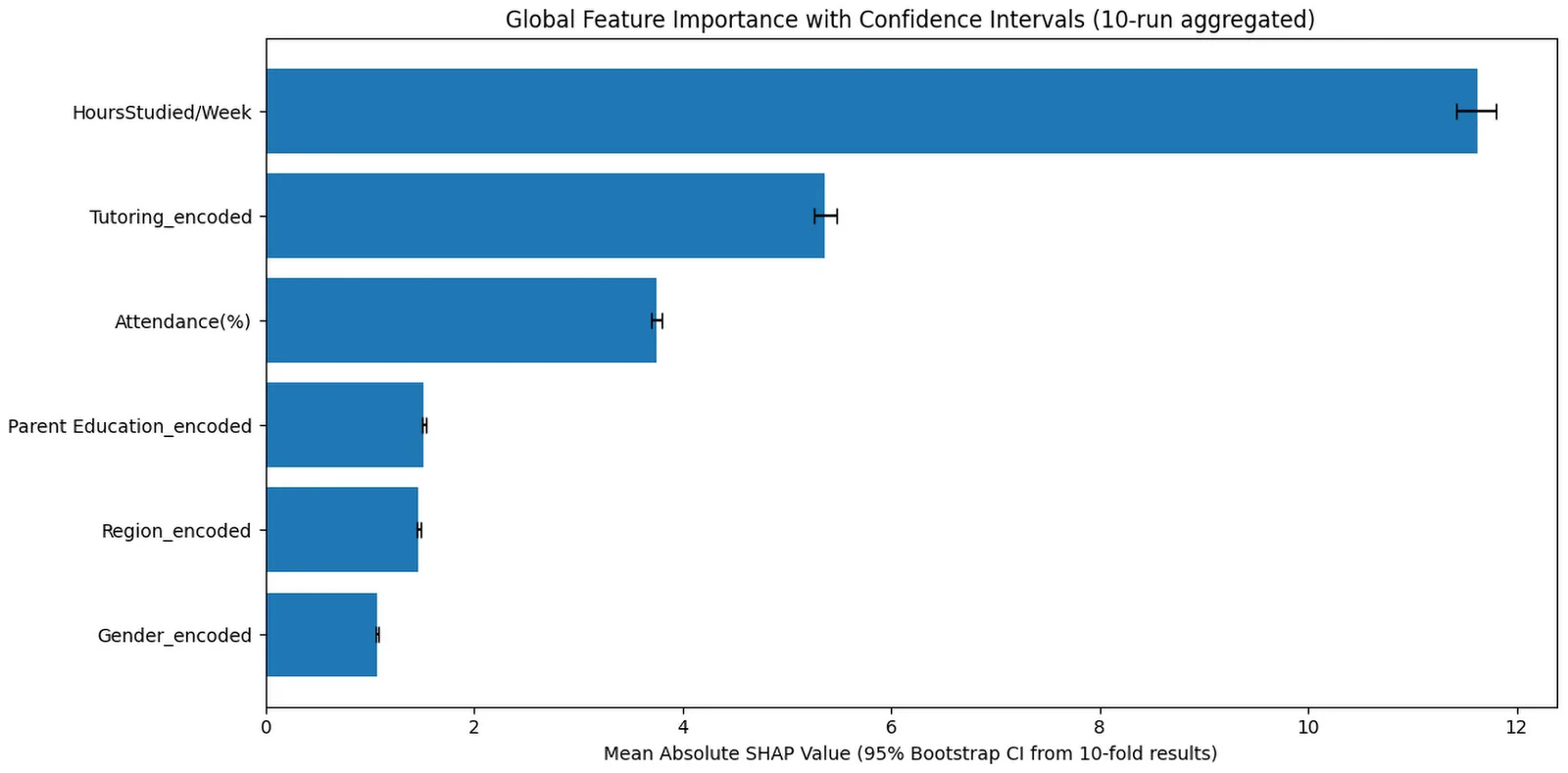
Global Feature Importance Rankings with 95% Bootstrap Confidence Intervals. Mean absolute SHAP values computed from aggregated SHAP values across all test samples from 10 independent train-test splits.

The SHAP analysis in this study provides three distinct contributions to educational data mining research. First, it quantifies the relative importance of different student-level predictors within a high-performance, non-linear model—going beyond traditional regression coefficients by capturing complex interactions and non-linear effects. Second, it reveals the direction and magnitude of each feature’s effect on predicted exam scores, offering actionable insights for educators. Third, it enables individual-level explanations (waterfall plots) that decompose each student’s prediction into feature-specific contributions, supporting personalized intervention strategies. These contributions are detailed below. Fig 6 presents the global feature importance rankings with 95% bootstrap confidence intervals, computed from aggregated SHAP values across all test samples from 10 independent train-test splits (approximately 8,000 samples in total). Among all features, HoursStudied/Week exhibits the highest mean absolute SHAP value (approximately 11.5), substantially exceeding all other features, indicating that weekly study time is the most critical factor influencing student exam scores. Tutoring_encoded (approximately 5.3) and Attendance(%) (approximately 3.8) rank second and third, respectively. In contrast, Region_encoded (approximately 1.5) and Gender_encoded (approximately 1.0) demonstrate relatively low importance.

HoursStudied/Week shows relatively narrow error bars, suggesting that its importance ranking remains highly consistent across different data splits. By comparison, features with lower importance (e.g., Gender_encoded, Region_encoded) exhibit wider confidence intervals, indicating greater uncertainty in their contributions. Consistent with prior findings in educational research (e.g., study time as a key predictor [10]; study hours and attendance [51]), our SHAP analysis reaffirms these factors as dominant predictors. However, our study extends these findings in two ways. First, by using SHAP within a non-linear model (TabPFN), we quantify the marginal effects of these features while accounting for complex interactions—something traditional linear regression models cannot fully capture. Second, the SHAP values provide a direct ranking of feature importance that is model-consistent and locally faithful, offering more reliable interpretations than coefficients from linear models or feature importance from tree-based models alone. This study contributes to the educational data mining literature by providing SHAP-based interpretability for TabPFN predictions, a combination that has not been previously examined in educational contexts.

**Fig 7:**
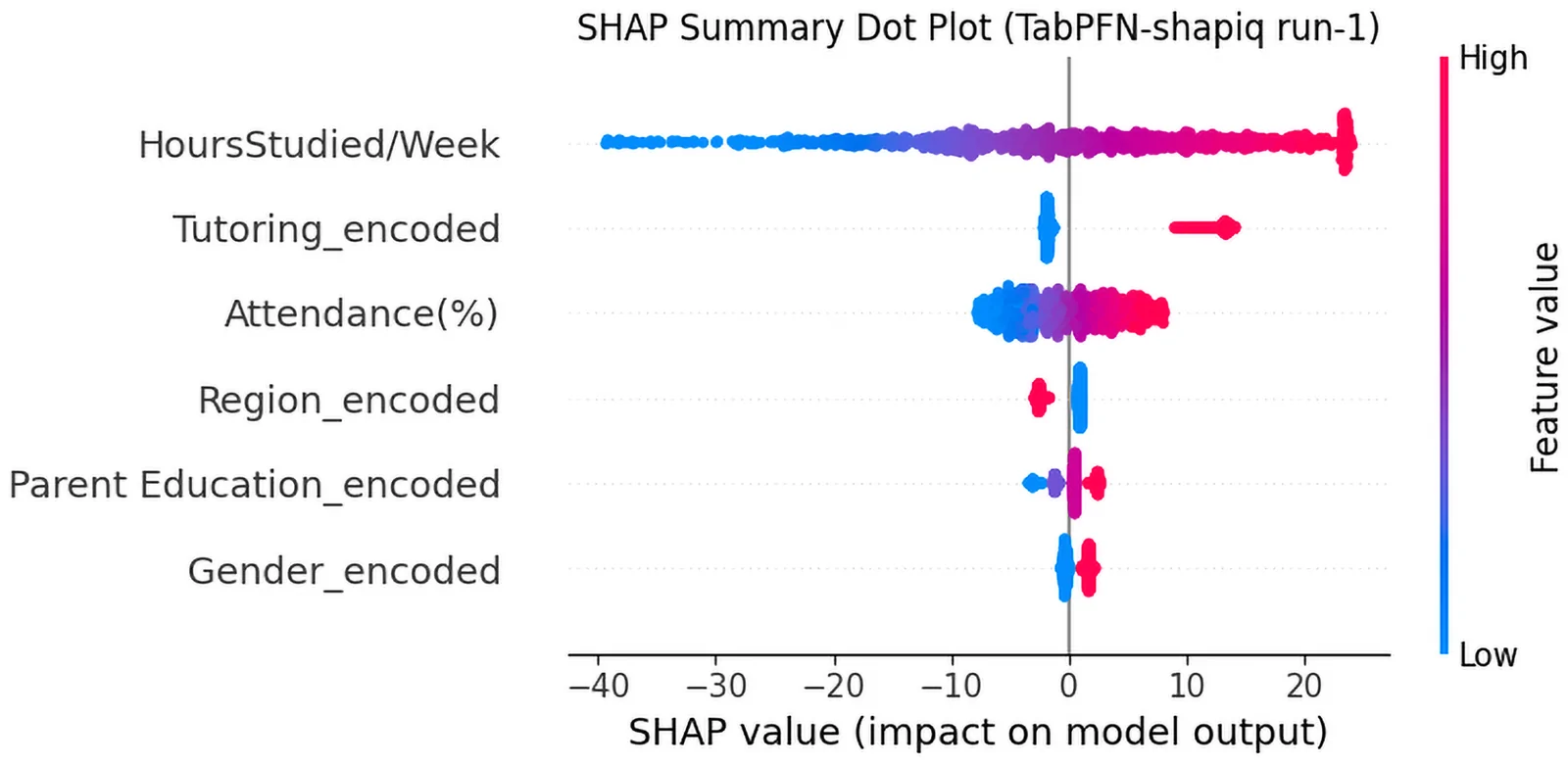
SHAP Summary Dot Plot. Impact of each feature on the TabPFN model output. The horizontal axis represents SHAP values; color gradient indicates feature values (red for high, blue for low).

Fig 7 presents a dot-style summary plot illustrating the direction and distribution of each feature’s impact on model output. The horizontal axis represents SHAP values (impact on model output), the vertical axis lists features in descending order of importance, and the color gradient indicates feature values (red for high, blue for low).

For HoursStudied/Week, higher study time (red points) corresponds to positive SHAP values, while lower study time (blue points) corresponds to negative SHAP values, exhibiting a clear monotonic positive relationship that further confirms the beneficial effect of study time on exam scores. Attendance(%) demonstrates a similar positive pattern, though with a relatively smaller magnitude of impact. Tutoring_encoded shows a bidirectional distribution—students who received tutoring (encoded as 1) exhibit mixed contributions to the prediction, suggesting that the effect of tutoring may be moderated by other factors. In contrast, the SHAP values for Gender_encoded and Region_encoded are dispersed and clustered near zero, indicating that gender and region contribute minimally and inconsistently to score prediction.

### Local explanation

**Fig 8:**
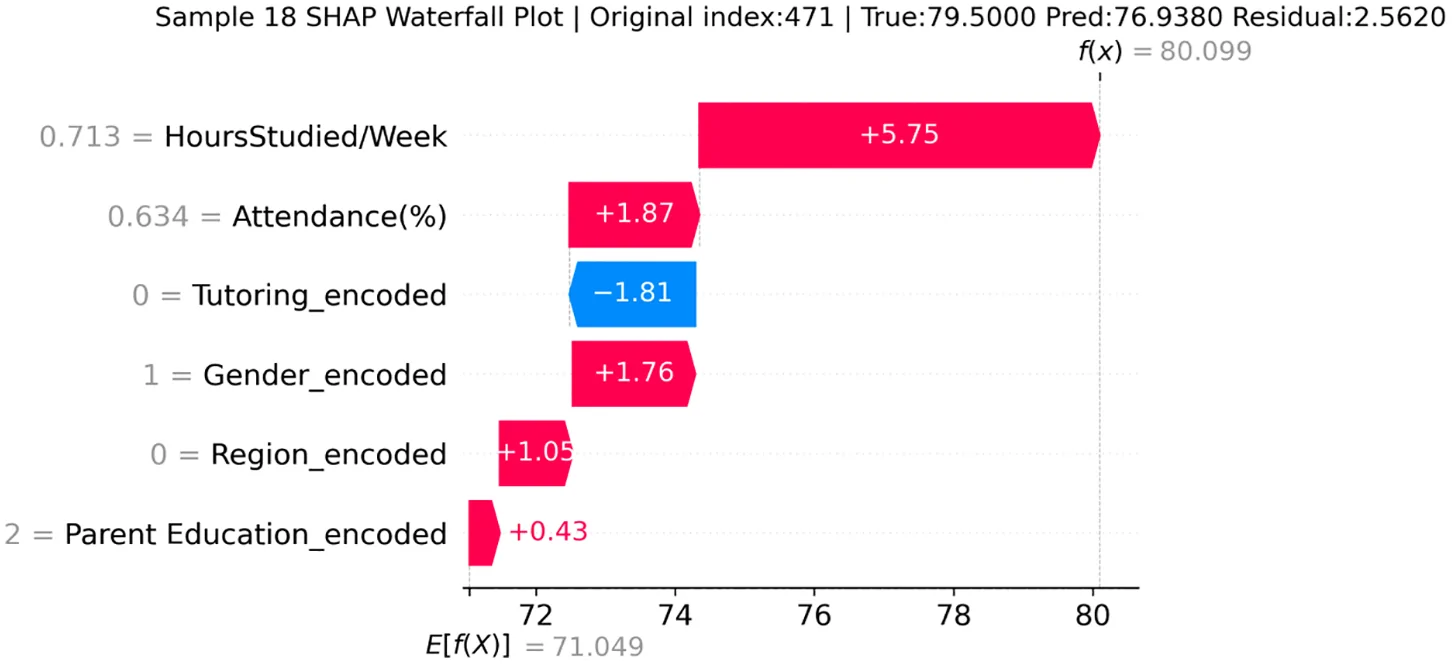
SHAP Waterfall Plot for an Individual Student Prediction (Sample 18, Original Index: 471). The plot decomposes the model’s prediction from the baseline value (71.05) to the final predicted score (76.94), showing each feature’s contribution.

Fig 8 presents the SHAP waterfall plot for Sample 18 (Original Index: 471) from the test set. The true exam score for this student was 79.50, the model predicted 76.94, with a residual of 2.56. The baseline value (i.e., the average prediction across the test set, E[f(X)]) was 71.05.

The decomposition of feature contributions for this prediction is as follows. HoursStudied/Week (feature value = 0.713) contributed the largest positive effect (SHAP = +5.75), raising the prediction from the baseline to approximately 76.80. Attendance(%) (feature value = 0.634) contributed a positive effect of +1.87, further increasing the predicted score. Gender_encoded (feature value = 1, indicating female) and Region_encoded (feature value = 0, indicating rural) contributed moderate positive effects of +1.76 and +1.05, respectively. Parent Education_encoded (feature value = 2, indicating secondary education) contributed a modest positive effect (+0.43). The sole negative contribution came from Tutoring_encoded (feature value = 0, indicating no tutoring, SHAP = −1.81). After aggregating all feature contributions, the final predicted score was 76.94.

This local explanation example illustrates how the TabPFN model generates personalized score predictions based on individual students’ multidimensional characteristics. For this particular student, study time, attendance, and gender jointly exerted positive effects, while the absence of tutoring contributed a moderate negative influence. Such individual-level transparent explanations provide actionable feedback for educators—when encountering students with similar feature profiles, priority attention may be given to their study time investment.

## Conclusion and Discussion

This study introduced the TabPFN algorithm, a pre-trained Transformer-based model, for predicting student academic performance in secondary education, and integrated the SHAP interpretability framework to enhance model transparency. Using the SAP-4000 dataset (4,000 Spanish high school students), we conducted a comprehensive comparative analysis against seven traditional machine learning models.

Our findings can be summarized as follows. First, TabPFN demonstrated superior predictive performance on the full dataset, achieving an RMSE of 4.8454, an R^2^ of 0.9216, and an MAE of 3.7950, outperforming all baseline models including XGBoost and LightGBM. Second, through systematic experiments across sample sizes ranging from 500 to 3,500 with 30 repeated random samplings, TabPFN exhibited stable performance even with limited data, achieving competitive accuracy at 500 samples that was comparable to or better than what traditional models attained at 3,500 samples. Third, SHAP analysis with 95% bootstrap confidence intervals, computed from 10 independent train-test splits, identified weekly study hours, tutoring, and attendance as the most influential predictors of academic performance, while region and gender contributed minimally.

These results suggest that TabPFN offers a promising approach for educational prediction tasks where data collection is often constrained by practical limitations. Its data efficiency and inherent suitability for tabular data make it particularly applicable in institutional research contexts with limited sample sizes. However, the performance gap between TabPFN and traditional ensemble methods narrowed as sample size increased, indicating that the choice of model should depend on the specific data availability of each application scenario. Traditional methods such as XGBoost and LightGBM remain viable alternatives when sufficient data are available.

Several limitations of this study should be acknowledged. First, while the additional case study on a distinct student performance dataset confirmed the framework’s generalizability, the primary dataset is limited to Spanish secondary education context; further validation across diverse educational systems and cultural settings is needed.. Second, the SHAP analysis, while providing valuable interpretability, offers approximate explanations that may be sensitive to the choice of explainer; future research could explore alternative interpretability methods such as LIME or rule extraction approaches. Third, although TabPFN demonstrates advantages in small-sample scenarios, its computational cost during inference is higher than that of traditional models, which may be a consideration for real-time applications.

Future research directions include: (1) validating the framework on larger and more diverse educational datasets, (2) extending the analysis to incorporate additional student-level and institutional-level features, (3) developing real-time intervention systems based on the predictive framework, and (4) exploring the integration of TabPFN with other emerging machine learning techniques for educational applications.

In summary, this study contributes to the growing body of educational data mining research by introducing and validating TabPFN as a robust and interpretable tool for academic performance prediction. The framework’s ability to achieve competitive accuracy with limited data, combined with SHAP-based transparency, provides a foundation for developing trustworthy AI systems that support personalized educational interventions and data-driven decision-making in resource-constrained environments.

## Supporting information

S1 Table Hyperparameter search spaces and optimal configurations for the evaluated machine learning models. The table lists the hyperparameter search space and the optimal configuration identified for each model. SVR, support vector regression; KNN, k-nearest neighbors; GBM, gradient boosting machine; Random Forest, random forest; XGBoost, extreme gradient boosting; LightGBM, light gradient boosting machine; TabPFN, tabular prior-data fitted network.
(XLSX)

S2 Table Training and inference times of the evaluated machine learning models. Training and inference times are reported in seconds. SVR, support vector regression; KNN, k-nearest neighbors; Ridge, ridge regression; GBM, gradient boosting machine; Random Forest, random forest; XGBoost, extreme gradient boosting; LightGBM, light gradient boosting machine; TabPFN, tabular prior-data fitted network.
(XLSX)

S3 Table Wilcoxon signed-rank test results comparing TabPFN with baseline models. The table reports the Wilcoxon statistic, p-value, and significance for each pairwise comparison between TabPFN and each baseline model. ** indicates p < 0.01. SVR, support vector regression; KNN, k-nearest neighbors; Ridge, ridge regression; GBM, gradient boosting machine; Random Forest, random forest; XGBoost, extreme gradient boosting; LightGBM, light gradient boosting machine; TabPFN, tabular prior-data fitted network.
(XLSX)
